# Evolution of TERT-interacting lncRNAs: expanding the regulatory landscape of telomerase

**DOI:** 10.3389/fgene.2015.00277

**Published:** 2015-09-10

**Authors:** Andrew D. L. Nelson, Dorothy E. Shippen

**Affiliations:** ^1^School of Plant Sciences, University of Arizona, Tucson, AZ, USA; ^2^Department of Biochemistry and Biophysics, Texas A&M University, College Station, TX, USA

**Keywords:** telomerase, TER, lncRNA, evolution, *Arabidopsis*

## Abstract

Long non-coding RNAs (lncRNAs) evolve rapidly and are functionally diverse. The emergence of new lncRNAs is driven by genome disturbance events, including whole genome duplication, and transposition. One of the few lncRNAs with a conserved role throughout eukaryotes is the telomerase RNA, TER. TER works in concert with the telomerase reverse transcriptase (TERT) to maintain telomeres. Here we discuss recent findings from *Arabidopsis thaliana* and its relatives illustrating the remarkable evolutionary flexibility within TER and the potential for non-canonical TERT-lncRNA interactions. We highlight the two TERs in *A. thaliana*. One is a conventional telomerase template. The other lncRNA negatively regulates telomerase activity in response to DNA damage, a function mediated by co-option of a transposable element. In addition, we discuss evidence for multiple independent TER loci throughout the plant family Brassicaceae, and how these loci not only reflect rapid convergent evolution, but also the flexibility of having a lncRNA at the core of telomerase. Lastly, we discuss the propensity for TERT to bind a suite of non-templating lncRNAs, and how such RNAs may facilitate telomerase regulation and off-telomere functions.

## Introduction

A major breakthrough in biology was the discovery that much of eukaryotic genomes are transcribed, yet only a small fraction of the transcripts derive from protein-coding genes. Most transcripts are long non-coding RNAs (lncRNAs). Generated from what were originally believed to be “dark” regions of the genome, lncRNAs number in the thousands to tens of thousands. Although only a few lncRNAs have been assigned a biological function, these molecules play essential roles in epigenetic regulation, stem cell biology and signal transduction and are emerging as important targets in human disease (Lee et al., 1996; Guttman et al., 2011; Wapinski and Chang, 2011; Scheuermann and Boyer, 2013). The molecular mechanisms of lncRNAs are varied, but appear to fall into four major categories: (1) molecular signals, (2) molecular decoys, (3) guides, and (4) scaffolds (Wang and Chang, 2011).

One of the best-studied lncRNAs is TER, the telomerase RNA. TER can be defined as a scaffolding lncRNA as it assembles into a ribonucleoprotein complex with several proteins including the reverse transcriptase TERT. TERT reiteratively copies a templating sequence embedded in TER to establish and maintain telomere repeats on chromosome ends. In stem and germline cells telomerase must continually replenish telomeric DNA to avoid cellular senescence, but in cells with limited proliferation programs the enzyme is repressed to avert tumorigenesis (Bernardes de Jesus and Blasco, 2013; Günes and Rudolph, 2013). Telomerase must also be precluded from acting at double-strand breaks (DSBs) to promote faithful DNA repair. Consequently, telomerase is subjected to multiple levels of regulation that target both TERT and TER (Cifuentes-Rojas and Shippen, 2012; Egan and Collins, 2012).

TER is highly variable in nucleotide sequence and size, ranging from ∼150 nucleotides in some ciliates to more than 1.2 kb in budding yeast (Egan and Collins, 2012). Despite its sequence variability, TER harbors conserved secondary and tertiary structures that are critical for TERT interaction and telomerase catalysis. These elements include a single-stranded region bearing the telomere template and a template boundary element that demarcates the 5′ end of the template. TERT binding is mediated by a pseudoknot adjacent to the telomere template (Zhang et al., 2011; Egan and Collins, 2012) and a stem terminus element (STE; Blackburn and Collins, 2011). Notably, the TER-TERT interaction does not require an intact telomere template, leaving open the opportunity for alternative lncRNAs to assemble into an RNP complex with TERT.

Although TERT and TER are sufficient to reconstitute telomerase enzyme activity *in vitro*, the essential domains of TER can be whittled down to a “Mini T” consisting of only ∼150 nts (Chen and Greider, 2003; Zappulla et al., 2005; Cifuentes-Rojas et al., 2011). Because most of the structural similarity within eukaryotic TERs lies within these 150 nts, conforming to TERT’s catalytic needs is a primary driver of TER conservation. TER assembles with suite of telomerase accessory proteins besides TERT that promote RNP maturation, modulate enzyme activity and facilitate telomerase recruitment to chromosome ends. More divergent than TERT, the accessory proteins typically are not shared between the major eukaryotic lineages (Collins, 2006). The ability of TER to accommodate a dynamic array of protein binding partners and yet retain its templating capacity demonstrates the advantage of having a lncRNA at the heart of the telomerase enzyme.

## The Impact of Genome Dynamics on lncRNA Evolution

**FIGURE 1:**
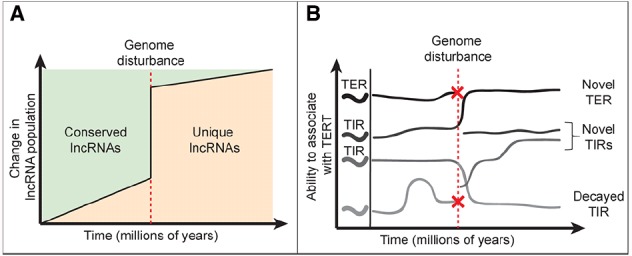
**Impact of genome dynamics on lncRNA evolution and TIR populations. (A)** Model for lncRNA evolution. Normally, lncRNAs evolve gradually due to accumulation of nucleotide changes and localized genome rearrangement events. However, genome disturbance events (red dashed line) accelerate lncRNA evolution leading to decay or loss of conserved lncRNAs and birth of new lncRNAs. **(B)** Impact of genome disturbance on TERT interacting RNA populations. Within the pool of lncRNAs that bind TERT, TER likely remains stable (as seen in vertebrates). Non-canonical TERT-interacting RNAs (TIRs) are likely to be more dynamic, moving into and out of the pool over time (decaying TIRs). The canonical TER remains stable until a genome disturbance event occurs (red dashed line), where the possibility of TER loss is high. If the ancient TER locus is lost (red X), another lncRNA, presumably a TIR, will replace it as the templating telomerase RNA. A genome disturbance event can also lead to novel lncRNA emergence **(A)**, whereby some of these RNAs may become TIRs.

TER, like other lncRNAs, does not harbor an open reading frame and thus can readily absorb nucleotide changes without a cost to fitness (Ponting et al., 2009; Kutter et al., 2012). Indeed, lncRNAs evolve rapidly and their evolution is influenced by factors besides the accumulation of nucleotide changes. Referred to here as genome disturbance events, whole genome duplication (WGD), genome rearrangement, and transposition all contribute to the volatility of lncRNA repertoires in eukaryotes (Freeling et al., 2012; Kapusta et al., 2013). Studies in vertebrates suggest that as genomes evolve, the lncRNA population slowly changes due to accumulation of nucleotide changes and local rearrangements (Figure 1A). In contrast, a genome disturbance event can trigger a dramatic spike in the emergence of novel lncRNAs and decay of more ancient ones. Following WGD duplicated chromosomes undergo a process called fractionation, whereby genes and whole genomic regions accumulate mutations and decay at a rapid rate (Freeling et al., 2012). While this process often leads to gene loss, pseudogenization or promoter acquisition can give rise to novel lncRNAs (Ponting et al., 2009). Genome disturbances are associated with rapid changes in lncRNA populations. Vertebrate genomes have remained relatively stable, and sequence orthologs for 20% of human lncRNAs are found in mice, including TER (Chen et al., 2000; Ponting, 2008; Necsulea et al., 2014). In contrast, less than 1% of *Arabidopsis thaliana* lncRNAs are evident in grape and poplar, two species with similar divergence times as that of human and mouse (Liu et al., 2012). The dramatic difference in identifiable lncRNA orthologs highlights the WGD and genome rearrangements that separate these plant species, and are consistent with the dynamic nature of plant genomes in general (Koenig and Weigel, 2015).

Transposable elements (TE) represent another means by which lncRNAs originate and diversify in vertebrates (Kapusta et al., 2013; Hoen and Bureau, 2015). Transposition can activate transcription adjacent loci, resulting in the birth of novel lncRNAs. TEs can also become incorporated into exons of lncRNAs in a process termed exaptation (Hoen and Bureau, 2015). TEs account for more than 30% of total lncRNA sequence. Moreover, roughly 70% of vertebrate lncRNAs contain at least some trace of repetitive elements. Unlike typical TEs that are silenced by cellular machinery, exapted elements may impart novel functions as well as contribute to integral facets of lncRNA maturation, such as transcription initiation, splicing, and polyadenylation (Keren et al., 2010; Kapusta and Feschotte, 2014). Additionally, exapted TEs are a common source of lineage-specific differential gene regulation (Lowe and Haussler, 2012). Johnson and Guigó (2014) argue that TEs have the potential to act as pre-formed functional RNA domains, endowing binding sites for novel interaction partners. For instance, TEs within XIST stimulate interactions with PRC2 and splicing factor ASF2 (Wutz et al., 2002; Jeon and Lee, 2011). As discussed below, TE exaptation into TER has dramatically influenced telomerase regulation in *A. thaliana*.

Given the volatile environment in which lncRNAs evolve, it is not surprising that TERs from different eukaryotic lineages bear little similarity to one another in both sequence and synteny (Chen et al., 2000; Cifuentes-Rojas et al., 2011; Qi et al., 2013). TERs from the major lineages likely represent convergent evolution, where unique and unrelated TERT-interacting RNA (TIR) molecules were adapted for use by the much more conserved TERT protein (Figure 1B). Despite their unique origins and disparate sequences, TERs from across much of eukarya have adapted similar core structural motifs and all require the templating domain in order to perform a very basic and conserved function: chromosome end maintenance (Chen et al., 2000; Qi et al., 2013).

## Brassicaceae as a System for Comparative lncRNA and Telomere Analyses

Recent data from the plant kingdom is providing unanticipated new insights into TER evolution. Beginning with Barbara McClintock’s pioneering work on maize telomeres in the 1930s (McKnight et al., 2002), plants have served as important models for chromosome biology. Their remarkable tolerance to genome instability and frequent WGD makes plants an important counterpoint to mammalian systems for analysis of genome dynamics and evolution. Brassicaceae is the most tractable of plant families and consequently the most valuable resource for comparative genomics. A large and diverse cadre of ∼3600 species, Brassicaceae grows throughout the world’s temperate zones and is believed to have arisen ∼65 mya (Koenig and Weigel, 2015). Brassicaceae is home to many agriculturally important plants species, but the most well-known member is *A. thaliana*. Due to its powerful genetics, *A. thaliana* has become the reference species for all plant biology (Jones et al., 2008), and has served as a model for telomere analysis for over 15 years (Watson and Riha, 2010).

The *A. thaliana* genome is compact (130 mb), yet is characterized by three rounds of WGD. The most recent occurred at the base of the family (Koenig and Weigel, 2015). The speciation event that gave rise to *A. thaliana* was followed by genome rearrangement and a reduction in chromosome number. Several other lineages within Brassicaceae have undergone WGD, and chromosome painting reveals a litany of large-scale chromosomal rearrangements (Mandáková and Lysak, 2008; Kagale et al., 2014). Thus, Brassicaceae and *A. thaliana* in particular serve as excellent systems for understanding how telomeres and telomerase components evolve in an ever-changing genomic environment.

Despite the dynamic nature of plant genomes, telomeric DNA has remained remarkably resistant to change. The telomere repeat sequence (TTTAGGG)_*n*_ is highly conserved throughout the plant kingdom, with a few interesting exceptions such as the order Asparagales (Sýkorová et al., 2003). Analysis of telomere length for twelve Brassicaceae species reveals some length variation, ranging from 850 bp to ∼9 kb (Nelson et al., 2014). However, this same degree of variation is observed among different ecotypes of *A. thaliana*, suggesting that factors modulating telomere length are conserved (Shakirov and Shippen, 2004). This conclusion is supported by the high degree of conservation associated with many telomere components [e.g., Cdc13/Stn1/Ten1 (CST) and TRF-like proteins; Karamysheva et al., 2004; Song et al., 2008; Surovtseva et al., 2009; Leehy et al., 2013; Nelson et al., 2014].

## Duplication of TER: Adding to Nature’s Toolbox of Telomerase Regulatory Mechanisms

The identification of telomerase protein components in *A. thaliana* has been driven largely by the conservation of subunits such as TERT, dyskerin and POT1 (Fitzgerald et al., 1999; Shakirov et al., 2005; Surovtseva et al., 2007; Kannan et al., 2008). TER, however, remained elusive until only a few years ago when telomerase-associated RNAs were identified by brute-force enzyme purification. These experiments unexpectedly uncovered more than one TER (Cifuentes-Rojas et al., 2011). TER1 (748 nt) and TER2 (784 nt) each contain 1.5 copies of the plant telomeric repeat sequence embedded in a 220 nt segment of ∼90% identity. In TER2 the conserved region is interrupted by a 529 nt unique sequence, subsequently shown to be a small transposon (see below). The transposon and the 3′ terminus are removed from TER2 to generate a smaller isoform termed TER2s (Cifuentes-Rojas et al., 2012). All three TER isoforms (TER1, TER2, and TER2s) assemble with TERT to reconstitute telomerase activity *in vitro*, indicating that the core elements required for catalysis are located in the conserved regions.

**FIGURE 2:**
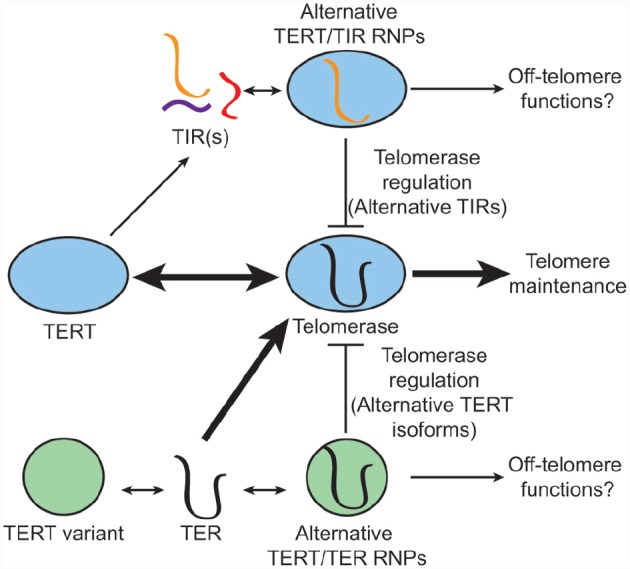
**Non-canonical telomerase subunits: alternative modes of enzyme regulation.** The conventional telomerase enzyme contains the core subunits TERT and TER (middle), which cooperate in telomere maintenance. TERT can also assemble with non-canonical TERT-interacting lncRNAs (TIRs) (top). Such RNAs may hijack the function of TERT, and in the case of *A. thaliana* TER2, inhibit telomerase activity by sequestering TERT in a non-productive RNP complex. Alternative TERT isoforms (bottom) have also been described. In humans, a major TERT splice variant has lost its catalytic activity, but retains TER binding. Like TER2, this non-canonical TERT is proposed to inhibit telomerase by sequestering TER. Non-canonical telomerase RNP complexes may also have alternative functions off the telomere.

Whereas the discovery of multiple TERs in *A. thaliana* was unusual, there is precedent for alternative telomerase subunits. Moreover, subpopulations of unassembled TERT and TER can be found in human cells (Xi and Cech, 2014), making the exchange and/or incorporation of non-canonical telomerase subunits feasible (Figure 2). The ciliated protozoan *Euplotes crassus* encodes three TERT proteins, which presumably assemble with a single TER, and act in different developmental stages to facilitate telomere maintenance during vegetative growth or *de novo* telomere formation during sexual development (Karamysheva et al., 2003). There are also variant TERT isoforms in humans, produced by alternative splicing (Ulaner et al., 2000, 1998; Saebøe-Larssen et al., 2006). A major splice variant (*β*-deletion) that is abundantly expressed in cancer and stem cells lacks the conserved reverse transcriptase domains, and yet retains TER binding. This variant behaves as a dominant negative inhibitor of telomerase (Figure 2). It can also protect against apoptosis in cancer cells, likely through a telomerase-independent mechanism (Listerman et al., 2013). A growing list of non-telomeric functions have been ascribed to TERT (Ale-Agha et al., 2014). The influence of lncRNA binding partners on such activities is unclear.

Variant TER isoforms have also been reported. Some appear to be processing intermediates (Chapon et al., 1997; Box et al., 2008). Others including the non-canonical TERs in pig and cow were proposed to be pseudogenes based on the presence of a mutation in the templating domain and deletions in other conserved domains (Chen et al., 2000). However, like hTERT splice variants, these alternative TERs have the potential to serve as dominant negative regulators or to play non-canonical roles in telomere biology (Figure 2).

A particularly interesting example of alternative TERs is found in *A. thaliana*, where TER gene duplication provided a fertile breeding ground for the appearance of a novel mode of telomerase regulation. TER1 is the canonical telomere template required for telomere maintenance in *A. thaliana* (Cifuentes-Rojas et al., 2011). TER2, by contrast, negatively regulates the TER1 RNP (Cifuentes-Rojas et al., 2012). Telomerase activity is elevated in *ter2* mutants, while TER2 over-expression reduces the TER1 templating function leading to telomere shortening. Conversely, mutation of the templating domain of TER2 does not cause incorporation of mutant telomere repeats on chromosome ends, indicating that TER2, despite its capacity to direct telomere repeat addition *in vitro*, does not productively engage chromosome ends *in vivo*. Notably, TER2 serves as a lncRNA scaffold for a different set of accessory proteins than TER1, which may contribute to its distinct function *in vivo* (Cifuentes-Rojas et al., 2011, 2012). Furthermore, TERT has a higher affinity for TER2 than for TER1. Thus, TER2 has the ability to serve as a molecular decoy or sponge that sequesters the telomerase catalytic subunit in a non-functional complex.

## Telomerase Regulation by Exaptation of a TE in TER

TER2 exhibits another of the lncRNA molecular paradigms: biological signal. Under standard growth conditions TER2 is a low abundance RNA, more poorly expressed than TER1 or TER2s (Cifuentes-Rojas et al., 2012). However, in response to DSBs, TER2 is rapidly induced and becomes the predominant TER isoform. Telomerase activity is repressed as TER2 levels rise. Remarkably, TER2 induction is not mediated by increased transcription, but rather by increased RNA stability (Xu et al., 2015). Thus, TER2 serves as a rapid regulatory switch linking the DNA damage response directly to telomerase enzyme activity.

Clues for how TER2 might function as a DNA damage sensor came from inspection of another unique feature of this molecule: its 529 nt intervening sequence (removed during the formation of TER2s). The intervening sequence contains no obvious branch point site, and the 5′ and 3′ splice sites do not match mRNA splicing consensus sequences. Instead the boundaries of this element consist of short inverted repeats flanked by two 5 nt direct repeats. Further analysis of similar sequences throughout Brassicaceae indicated that the intervening sequence within TER2 is in fact a small TE, a solo long terminal repeat from a gypsy class of retrotransposons (Xu et al., 2015).

A TE is associated with the majority of TER2 loci in *A. thaliana* ecotypes but not all, providing an opportunity to assess if and how this element modulates telomerase behavior. The unique behavior of TER2 appears to be largely, if not entirely dependent on its TE (Xu et al., 2015). Without the TE, TER2 is a highly stable lncRNA that binds TERT with a lower affinity than TER1. Moreover, in *A. thaliana* ecotypes lacking the TER2 TE, telomerase regulation by DSBs is lost. Thus, exaptation of a TE into the TER2 locus profoundly influenced the regulation and behavior of this lncRNA by endowing it with a DNA damage sensor and the capacity to sequester TERT in a non-productive complex. This mode of telomerase regulation is expected to promote genome stability and may be especially beneficial during meiosis when genome-wide DSBs abound.

## Evolution of TER as a TERT-associated lncRNA

Phylogenetic analysis, and particularly gene synteny, has revealed numerous lncRNAs orthologs, including TER, in several eukaryotic lineages (Chen et al., 2000; Qi et al., 2013). Beilstein et al. (2012) employed this strategy to identify an *A. thaliana* TER-like locus from 14 species sampling the breadth of Brassicaceae. However, three unanticipated findings were uncovered. First, *AtTER1* and *AtTER2* loci represent an *A. thaliana*-specific duplication event. In *A. lyrata*, the closest relative of *A. thaliana*, only a single *TER-like* locus was detected. Further analysis showed that the *A. thaliana TER1/TER2* duplication occurred as part of a large-scale genome rearrangement coinciding with *A. thaliana* speciation (Beilstein et al., 2012). Second, contrary to findings from yeast and mammals, there is no clear phylogenetic signature of conservation at the *TER-like* loci in Brassicaceae to infer critical structural and functional elements. The evolutionary pressures placed on each of these loci must be distinct. Third, and most surprisingly, the telomere templating domains of *TER-like* loci in multiple Brassicaceae species including *A. lyrata* carry point mutations that would preclude synthesis of TTTAGGG repeats. Hence, an alternative locus must encode the canonical TER in many Brassicaceae species.

The Brassicaceae TERs and TIRs provide a fascinating window into both the molecular mechanisms and evolution of lncRNAs. Indeed TER2’s emergence by TE exaptation may be only one example of how lncRNAs evolved to regulate TERT. We postulate that transformation of TER2 into a TERT decoy reflects TERT promiscuity for RNA. The ancient origin of TERT from a viral reverse transcriptase supports the notion that TERT evolved RNA specificity over time (Curcio and Belfort, 2007). Even now, sequencing of TIRs in human cells revealed >30 unique RNA species (Maida et al., 2009). In the event that a species’ canonical TER locus is lost, a replacement is likely adapted from the pool of TIRs (Figure 1B). Throughout the co-option of a novel TER, TERT would still have the capacity to assemble with a suite of non-templating TIRs and by extension their alternative accessory proteins. TIRs therefore have the potential to modulate conventional and non-conventional TERT-related activities (Figures 1B and 2). Consequently, this intriguing class of lncRNAs provides new insights into regulating telomerase and potentially other cellular functions in cancer and age-associated diseases.

### Conflict of Interest Statement

The reviewer Aaron Tarone declares that, despite being affiliated with the same institute as the author Dorothy Shippen, the review process was handled objectively. The authors declare that the research was conducted in the absence of any commercial or financial relationships that could be construed as a potential conflict of interest.
